# Intraocular Injection of StivantⓇ (A Biosimilar to Bevacizumab): A Case Series

**DOI:** 10.18502/jovr.v16i1.8248

**Published:** 2021-01-20

**Authors:** Ahmad Mirshahi, Alireza Lashay, Hamid Riazi-Esfahani, Nazanin Ebrahimiadib, Hassan Khojasteh, Fariba Ghassemi, Fatemeh Bazvand, Alireza Khodabande, Ramak Roohipour, Elias Khalili Pour, Hooshang Faghihi

**Affiliations:** ^1^Translational Ophthalmology Research Center, Farabi Eye Hospital, Tehran University of Medical Sciences, Tehran, Iran

**Keywords:** Stivant, Bevacizumab, Anti-VEGFs, Anti-vascular Endothelial Growth Factors, Diabetic Macular Edema, Retinal Vein Occlusion, Neovascular Age-related Macular Degeneration

## Abstract

**Purpose:**

To report the results of intravitreal injection of a bevacizumab biosimilar called StivantⓇ

**Methods:**

This prospective interventional case series was conducted on eyes with neovascular age-related macular degeneration (nAMD), retinal vein occlusion (RVO), and diabetic macular edema (DME). StivantⓇ was injected in three consecutive months and changes in best-corrected visual acuity (BCVA) and central macular thickness (CMT) were measured at baseline and monthly up to one month after the third injection.

**Results:**

Three hundred and eighty-five eyes with DME (234 eyes, 61%), nAMD (87 eyes, 22%), and macular edema secondary to RVO (64 eyes, 17%) were enrolled. The mean ± standard deviation age of the patients was 61.7 ± 7.20 years. The mean BCVA and CMT changed from 0.63 ± 0.3 to 0.51 ± 0.3 LogMAR (*P* = 0.12 ) and from 420.4 ± 47.3μm at baseline to 316.7 ± 50.6 μm (*P* < 0.001) in the DME group; from 0.79 ± 0.3 to 0.68 ± 0.3 LogMAR (*P* = 0.19) and from 376.1 ± 31.7 μm to 303 ± 31.3 μm (*P* = 0.019) in the nAMD group; and from 0.81 ± 0.4 to 0.63 ± 0.4 LogMAR (*P* = 0.05) and from 424.21 ± 18 μm to 303.4 ± 18.8 μm (*P* < 0.001) in the RVO group, respectively.

**Conclusion:**

Our limited experience showed that the intravitreal injection of StivantⓇ was well tolerated. Although the results of this case series showed relative improvement in CMT one month after the last injection of StivantⓇ, BCVA improvement was statistically significant only in the RVO group. This would be essential to design a randomized clinical trial to evaluate the non-inferiority of StivantⓇ in comparison to bevacizumab.

##  INTRODUCTION

Introduction of anti-VEGFs has revolutionized the management of numerous retinal diseases over the past decade. They turned out to be the first-line treatment for diabetic macular edema (DME), neovascular age-related macular degeneration (nAMD), and retinal vein occlusion (RVO)-associated macular edema. Among various anti-VEGF drugs, the off-label intravitreal injection of bevacizumab [Avastin; Genentech/Roche /Basel, Switzerland] as a less expensive and effective alternative is the preferred choice in many countries^[1]^.

Results of clinical trials such as CATT (Comparison of AMD Treatments Trials), MANTA (Multicentre Anti-VEGF Trial in Austria), IVAN (The Inhibition of VEGF in Age-related choroidal Neovascularization), LUCAS (Lucentis Compared to Avastin Study), and GEFAL (Groupe d'Etude Français Avastin versus Lucentis ) showed the noninferiority of bevacizumab in comparison to ranibizumab with the same safety profile^[2,3,4,5,6]^. Besides, the 20 times lower cost of bevacizumab compared to ranibizumab and aflibercept makes this agent the most common anti-VEGFs used for intravitreal injection^[7]^.

The World Health Organization (WHO) defines biosimilar drugs as a biotherapeutic product that is similar in terms of quality, safety, and efficacy to an already licensed reference biotherapeutic product. Biosimilars have the potential to reduce the healthcare costs relative to reference biologics, thereby increasing the treatment access [8–10]. StivantⓇ (CinnaGen Co., Iran) has been developed as a biosimilar to AvastinⓇ. Both StivantⓇ and the reference product are humanized monoclonal antibodies of the IgG1 subclass. Safety of this product has already been shown during an animal study conducted by our team on New Zealand albino rabbits. Intravitreal injection of 2.5 mg StivantⓇ did not show any adverse effect on retinal function evaluated by electroretinography (ERG). Additionally, histologic examination of the enucleated globes did not reveal any visible histopathologic changes at the cellular level^[8]^.

Herein, we aimed to share our experience with visual and anatomical outcomes of intravitreal injection of StivantⓇ in a case series.

##  METHODS

This prospective interventional case series was approved by the Institutional Review Board of Tehran University of Medical Sciences. Written informed consent was obtained from participants before enrollment. Patients with neovascular AMD (nAMD), DME, and macular edema due to RVO were recruited from September 2018 to February 2019 at Farabi Eye Hospital, Tehran, Iran. They were either treatment-naïve or had not receive the last intravitreal injection during the past six months.

The exclusion criteria of the study included previous vitrectomy, signs of any ocular infection, history of cerebrovascular accident or myocardial infarction, pregnancy, or breastfeeding. All patients were scheduled for three monthly injections of StivantⓇ.

Complete ocular examinations were performed by ophthalmologists and included measurement of best-corrected visual acuity (BCVA) with the Snellen chart being converted into LogMAR, applanation tonometry, slit-lamp biomicroscopy of the anterior and posterior segments, and indirect ophthalmoscopy at baseline and on days 1, 7, and 30 after each injection. Spectral-domain optical coherence tomography (SD-OCT) (RTVue-XR; Optovue, Inc., Fremont, CA, USA) imaging was obtained at baseline and 30 days after each injection for all patients.

Parameters for safety included severe inflammation or endophthalmitis and IOP > 21 mm Hg, retinal hemorrhages, retinal vasculitis, and retinal necrosis or detachment within three months post-injection. Systemic evaluations at baseline and on days 1, 7, and 30 included a detailed medical history during which patients were asked about current medications and any systemic adverse events (AEs), thromboembolic or neurological issues and measurement of arterial blood pressure. Primary outcome measures were changes in CMT and BCVA. Secondary outcome measures comprised any ocular or systemic AEs.

### Intravitreal Injection

StivantⓇ is manufactured in a vial with a concentration of 25 mg/ml identical to the reference product (Avastin). Intravitreal injections were performed in the operating room under the sterile situation. Topical anesthetic drops were given first and then a lid speculum was inserted. After the application of povidone iodine 5% into the conjunctival sac for about 3 min, intravitreal injection of 1.25 mg/ 0.05 ml StivantⓇ was performed with a 29-gauge needle (1 ml tuberculin syringes; DispoVan) through the pars plana 4 mm and 3.5 mm posterior to the limbus in phakic and pseudophakic eyes, respectively. The needle was carefully removed using a sterile cotton applicator to prevent reflux. Pre-injection topical antibiotics were not ordered, but all patients received topical chloramphenicol 0.5% four times a day for five days after the injection.

### Statistical Analysis

Data were entered into a Microsoft Excel sheet and analyzed using the SPSS version 22 software (IBM). Categorical data were represented in the form of frequencies and proportions. Chi-square was used as the test of significance. Continuous variables were summarized by count, mean, standard deviation, median, and minimum and maximum. BCVA and CMT data were analyzed using two-tailed paired *t*-tests. *P* ≤ 0.05 was considered statistically significant.

##  RESULTS 

Three hundred and eighty-five eyes of 351 patients with DME (234 eyes, 61%), nAMD (87 eyes, 22%), and macular edema secondary to RVO (64 eyes, 17%) were enrolled. Intravitreal injection of StivantⓇ from separate glass vials was performed in both eyes of 34 patients with bilateral DME. The mean age of the patients was 61.7 ± 7.20 years. Out of the 385 injections, 212 (55.1%) were performed in male patients. Of the 385 eyes, 197 and 188 were phakic and pseudophakic, respectively.

### BCVA Findings

**Figure 1:**
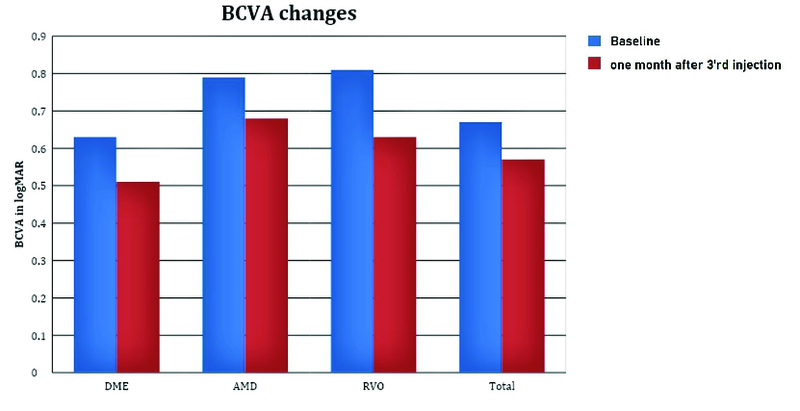
Mean BCVA (LogMAR) at baseline and one month after the third StivantⓇ injection. (*P*-value = 0.12, 0.19, 0.05, and 0.10 in the DME, wet-type AMD, RVO, and in all patients, respectively).

The mean BCVA improved from 0.67 ± 0.41 LogMAR at baseline to 0.57 ± 0.37 LogMAR one month after the last injection (*P* = 0. 10). The mean BCVA improved from 0.63 ± 0.3 to 0.51 ± 0.3 LogMAR (*P* = 0.12) in the DME group; from 0.79 ± 0.3 to 0.68 ± 0.3 LogMAR (*P* = 0.19) in the nAMD group; and from 0.81 ± 0.4 to 0.63 ± 0.4 LogMAR (*P* = 0.05) in the RVO group [Figure 1].

**Figure 2:**
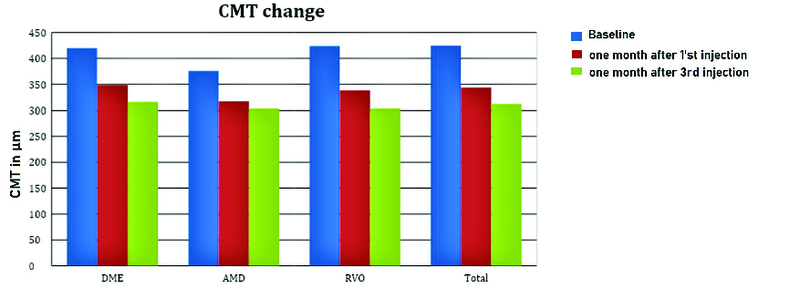
Central macular thickness (CMT) changes. Mean CMT ± SE (μm) at baseline and one month after the first and third StivantⓇ injection in the DME, AMD, RVO, and in all patients.

**Figure 3:**
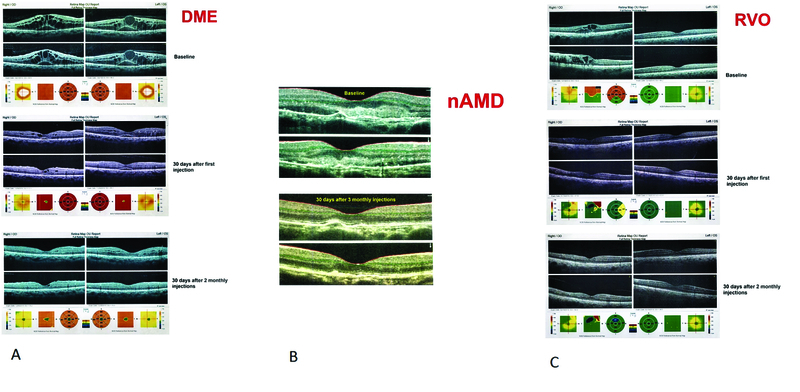
Response to StivantⓇ injections; samples from each subgroup (DME, nAMD, RVO) under study (A–C).

### Central Macular Thickness Findings

The mean CMT in all groups improved consistently from baseline through consequent injections. Although there was a trend in decreasing CMT after the first injection, the amount of change was not statistically significant until the third injection. The mean CMT of 425 ± 54.9 μm at baseline decreased to 312.20 ± 40.81 μm one month after the last intravitreal injection (*P* < 0.001) in all groups. In the DME group, the mean thickness decreased from 420.4 ± 47.3 μm at baseline to 316.7 ± 50.6 μm (*P* < 0.001) one month after the last intravitreal injection and from 376.1 ± 31.7 μm to 303 ± 31.3 μm (*P* = 0.019) in the nAMD group and from 424.21 ± 18 μm to 303.4 ± 18.8 μm (*P* < 0.001) in the RVO group [Figures 2 and 3].

### Adverse Events (AEs)

There was no reported drug-related blurred vision and/or ocular pain at any of the follow-up visits. None of the eyes developed intraocular inflammation, endophthalmitis, corneal edema, cataract, vitritis, retinal detachment, or optic atrophy. Vitreous hemorrhage was reported in a diabetic patient one day after injection, which resolved three weeks later. None of the patients experienced moderate or severe vision loss (>0.3 LogMAR). The mean IOP at day 30 was 16.1 ± 3.0 mmHg. No systemic or serious AEs were reported.

##  DISCUSSION

In the current case series, we showed the relative safety of intravitreal injection of a bevacizumab biosimilar (StivantⓇ) in eyes with different indications for anti-VEGF therapy.

Although the short-term results in the present study showed statistically significant improvement in terms of CMT reduction following intravitreal StivantⓇ injection in all three groups, the mean BCVA improvement reached statistical significance only in the RVO group. To demonstrate the substitutability of StivantⓇ as a biosimilar of Avastin, there is a need to design a randomized clinical trial (RCT) with an appropriate sample size.

We previously disclosed the safety of StivantⓇ during an animal study. This biosimilar did not show histopathologic changes at the cellular level after being injected into the eyes of albino rabbits evaluated by clinical examinations, ERG, and histopathological assessment.^[8]^

Biosimilars which are produced by modified cellular processes are identical to their reference biologic agents in terms of structure and active substance, although some minor variations are inevitable. Therefore, biosimilars are the end products similar to the original molecule with minor non-significant differences.^[9,10,11,12]^

In February 2015, RazumabⓇ (Intas Pharmaceuticals, Ahmedabad, India), the first biosimilar to ranibizumab, was approved by the drug controller general of India for the treatment of nAMD, DME, RVO-associated macular edema, and myopic choroidal neovascularization. In a prospective study, the safety and efficacy of RazumabⓇ was demonstrated in Indian patients with retinal vascular diseases including RVO.^[13,14]^ Afterward, Warudkar et al showed the safety and efficacy of intravitreal injection of Zybev (Cadila Healthcare, India) as a bevacizumab biosimilar for macular edema secondary to retinal vascular diseases.^[15]^ As the patent of Avastin has recently expired, it is speculated that its biosimilars will soon grow in number.^[10,11]^

Biosimilar production is >25% cheaper than that of the reference drug.^[10,11]^ As a result, more patients, especially in developing countries, can adhere to their treatment protocols and sustain their vision.

With the increasing production of biosimilar drugs in different countries and lower costs of these drugs compared to reference biologics, the widespread usage of these drugs requires special attention of healthcare systems to evaluate them from several aspects, including pharmacokinetics, pharmacodynamics, immunogenicity, safety, and efficacy in comparison to the reference drugs.^[11]^

While the main focus of the reference drug producer is to display safety and efficacy in large clinical trials, biosimilar expansion mainly relies on thorough studies to approve that the product is indistinguishable from reference drug in terms of construction, synthesis, and in vitro activity. As a minimum, one clinical investigation is required to compare the pharmacokinetics between a reference and biosimilar drug and at least one adequately large randomized controlled trial to exhibit the clinical equality.^[9][10,11]^

Safety and efficacy equivalency of the biosimilar drugs to the reference drug concerning pharmacokinetic, pharmacodynamic, and immunogenic properties must be confirmed through well-designed clinical trials. If the results of these trials are satisfactory and a biosimilar drug is approved for one indication, all other indications, for which the reference product is approved, are accepted, provided there is appropriate scientific justification. In general, patients are expected to be able to shift from a biosimilar to a reference product and vice versa without a drug efficacy lapse or increased risk.^[9,10]^

Recently, the US Food and Drug Administration (FDA) gave directions to address the extra administrative requirements that biosimilars need to be endorsed as compatible drugs, and has recommended patrons to conduct at least one switching investigation to exhibit that the biosimilar and the reference drug can be securely substituted without loss of efficacy. Interestingly, in the European Union (EU), the European Medicines Agency (EMA) has not assigned biosimilars as interchangeable substitution of a reference medicine, leaving the choice to national authorities.^[10]^

As mentioned previously, this study is just a case series of patients and our findings cannot replace a well-designed, controlled RCT to show the equivalency of StivantⓇ with the reference drug. The other limitations of our study are the short-term follow-up of four months and the lack of data on metabolic profiles such as HbA1C and blood pressure of enrolled diabetic patients.

In conclusion, our limited experience showed that the intravitreal injection of StivantⓇ was well tolerated over four months. Although the results of this case series showed relative improvement in CMT one month after the last injection of StivantⓇ, the mean BCVA improvement was statistically significant only in the RVO group. To evaluate the non-inferiority, safety, and efficacy of StivantⓇ in comparison to the reference drug, it is essential to design a randomized clinical trial.

##  Acknowledgement

The authors would like to thank Leila Buzh Abadi and Pouran Fadakar for their kind contribution to this study.

##  Financial Support and Sponsorship 

The study received support from the Eye Research Center, Farabi Eye Hospital, Tehran University of Medical Sciences, TUMS#40777.

##  Conflicts of Interest 

None of the authors have any proprietary interests or conflicts of interest related to this study.
